# Auricular Hemorrhage Following Suppression of the Menses

**Published:** 1846-08

**Authors:** 


*Auricular Hemorrhage following Suppression of the Menses.*—Such is the title of an interesting observation that M. Alibert of Castelnaudary has published in the *Journal de Médecine et de Chirurgie de Toulouse.*

Paule Encely of Saint Amans, aged 45 years, experienced nine years ago a sudden stoppage of the menses, following the application of cold to the feet, or agitation, and perhaps both. The menses have not appeared since that time, but she became deaf, and every month there flowed from the right ear an ounce and a half of blood. The discharge continued twenty-four or forty-eight hours, and ceased spontaneously. It was announced by certain premonitory symptoms, which custom had taught the woman indicated an approaching flow of blood, consisting of an inconvenient sense of weight in the head, noises, and a sensation as if numerous ants were buzzing in the ear affected. The patient herself observed the regular periodic nature of this discharge, and understood that it was supplied by nature to compensate for the absence of the menses.

At present, this loss of blood no longer retains its menstrual type. It came back at indeterminate intervals, and differs more or less from the quantity formerly discharged. This irregularity is accompanied by violent cephalalgia, fugitive vertigo, dimness of sight, and, in short, a congestive state of the brain. The general health is otherwise perfect.—*Ibid, from Encyclographie Médicale.*

